# Internal transcribed spacer 2 (ITS2) barcodes: A useful tool for identifying Chinese *Zanthoxylum*

**DOI:** 10.1002/aps3.1157

**Published:** 2018-06-15

**Authors:** Li‐Li Zhao, Shi‐Jing Feng, Jie‐Yun Tian, An‐Zhi Wei, Tu‐Xi Yang

**Affiliations:** ^1^ College of Forestry Northwest A&F University Yangling Shaanxi 712100 People's Republic of China; ^2^ Research Centre for Engineering and Technology of *Zanthoxylum* State Forestry Administration Yangling Shaanxi 712100 People's Republic of China; ^3^ College of Life Science Northwest A&F University Yangling Shaanxi 712100 People's Republic of China

**Keywords:** DNA barcoding, ETS, ITS2, Rutaceae, *trnH‐psbA*, *Zanthoxylum*

## Abstract

**Premise of the Study:**

The genus *Zanthoxylum* in the Rutaceae family of trees and shrubs has a long history of domestication and cultivation in Asia for both economic and medicinal purposes. However, many *Zanthoxylum* species are morphologically similar and are easily confused. This often leads to false authentication of source materials and confusion in herbal markets, hindering their safe utilization and genetic resource conservation. DNA barcoding is a promising tool for identifying plant taxa.

**Methods:**

We used three candidate DNA barcoding regions (ITS2, ETS, and *trnH‐psbA*) to identify 69 accessions representing 13 Chinese *Zanthoxylum* species. The discriminatory capabilities of these regions were evaluated in terms of PCR amplification success, intra‐ and interspecific divergence, DNA barcoding gaps, and identification efficiency using the BLAST and tree‐building methods.

**Results:**

ITS2 proved the most useful for discriminating Chinese *Zanthoxylum* species, with a correct identification rate of 100%, and this region also exhibited significantly higher intra‐ and interspecific divergence.

**Discussion:**

Phylogenetic analysis confirmed that ITS2 has a powerful discriminatory ability both at and below the species level. We confirmed that ITS2 is a powerful barcoding region for identifying Chinese *Zanthoxylum* species, and will be useful for analyzing and managing Chinese *Zanthoxylum* germplasm collections.

*Zanthoxylum* L., belonging to the Rutaceae family, is a genus of ~250 species of evergreen and deciduous shrubs, trees, and woody climbers primarily distributed in tropical and subtropical regions worldwide, with 39 species and 14 varieties in China (Patiño et al., 2008). *Zanthoxylum* has a long history of cultivation and domestication in Asia for both economic and medicinal purposes. Plants in this genus are a resource for alkylamides (Bryant and Mezine, 1999; Yang, 2008), which satisfy the human numb‐taste sensation and serve as traditional medicines. The Chinese herbology resource *Compendium of Materia Medica* (also known as *Bencao Gangmu*) reports that *Z. bungeanum* Maxim. is effective for the treatment of toothache, diarrhea, and rheumatism (Wei et al., 2012). According to recent research, compounds isolated from the roots of *Z. planispinum* Siebold & Zucc. displayed potential anticancer activity (Su et al., 2015). Moreover, Szechuan peppers (commonly derived from *Z. armatum* DC. and *Z. bungeanum*) are widely consumed as a spice among the “eight essential condiments” in the kitchen due to their exceptional aroma and flavor (Li et al., 2016). In addition, the cultivated species *Z. bungeanum* and *Z. armatum* are often used as mountain ecological afforestation trees under China's “Grain for Green” program (Cheng et al., 2015).

Traditionally, identification of *Zanthoxylum* species has mainly been based on morphological characteristics such as fruit color and leaf shape (Tu et al., 2001). However, identification of some species of *Zanthoxylum* is difficult when based solely on morphological characteristics, which are often affected by growth habitats. For example, the cultivar ‘Qinghuajiao’ is often mistaken for *Z. schinifolium* Siebold & Zucc., but is actually *Z. armatum* (Feng et al., 2015). Furthermore, some cultivars or varieties that share similar morphological characteristics often bear the same names, but actually belong to different species. The incorrect selection of cultivars in agricultural production can result in economic losses; therefore, it is necessary to apply techniques such as DNA barcoding to the *Zanthoxylum* genus for discrimination.

DNA barcoding is a molecular diagnostic technique in which species identification is performed by using a short, standardized DNA region (Hebert et al., 2003, 2004). The cytochrome c oxidase subunit I (COI) gene has been widely found to have a high ability to differentiate between animal species (Hebert et al., 2003). However, in plants, the low mutation rate of the COI gene has led to the search for alternative barcoding regions (Chase et al., 2005; Kress et al., 2005; CBOL Plant Working Group, 2009), and studies have focused on chloroplast and nuclear genes (Cowan et al., 2006; Pennisi, 2007). Many loci have been proposed as plant barcodes. Although no consensus has emerged, different research groups have embraced DNA barcoding as a practical tool for taxa identification and have proposed various different loci as preferred plant barcodes (Pennisi, 2007; Erickson et al., 2008; Lahaye et al., 2008). For example, the Consortium for the Barcode of Life Plant Working Group (CBOL) evaluated seven plastid regions for land plants and proposed the two‐locus *rbcL*+*matK* as a universal plant DNA barcode (CBOL Plant Working Group, 2009). However, because *rbcL* has low sequence variation (Chen et al., 2010) and *matK* has low primer universality (Sass et al., 2007; Piredda et al., 2011; Kool et al., 2012), this two‐locus barcode is far from perfect, and the search for a more appropriate DNA barcoding region for plants is ongoing.

In the nuclear genome, the ITS gene, especially ITS2, is a potential DNA barcode, and it has been used in multi‐loci barcode combinations (Chase et al., 2005; Feng et al., 2016a). Schultz et al. (2005) advocated that ITS2 has potential as a general phylogenetic marker. Chen et al. (2010) tested the discriminatory ability of ITS2 in more than 6600 plant samples belonging to 4800 species from 753 distinct genera, and proposed that it can be used as a standard DNA barcode to identify medicinal plants. ITS2 has since been shown to be a promising and effective region for identifying medicinal samples (Feng et al., 2016a; Han et al., 2016).

The ETS region is often used for phylogenetic analysis (Li et al., 2007). Acevedo‐Rosas et al. (2004) used the ETS region to determine relationships among species of *Graptopetalum* Rose and closely related genera, and found that it had the highest number of parsimony‐informative sites. Li et al. (2002) used the ETS sequence to examine paraphyletic relationships in *Syringa* L. and *Ligustrum* L., and Mo (2015) tested the ETS sequence in *Paulownia* Siebold & Zucc. and confirmed it as a candidate DNA barcode for this genus.

The chloroplast genome, particularly the *trnH‐psbA* region, is one of the more variable intergenic spacers in plants and can be recommended as a supplementary barcode (Hollingsworth et al., 2011). Kress et al. (2005) proposed that the *trnH‐psbA* plastid spacer region is suitable for DNA barcoding of flowering plants, and it was used in a two‐locus barcode system. The *trnH‐psbA* region was later used for a wide range of plants, and an increasing number of researchers have proposed it as a supplementary DNA barcode for plant taxa (Newmaster et al., 2008; Chen et al., 2010).

Because identifications based on molecular markers are independent of environmental variation, molecular markers have been popular tools for modern taxonomists due to their stability and universality (Feng et al., 2016a). Some molecular markers, including simple sequence repeat (SSR) and sequence‐related amplified polymorphism (SRAP) markers, have been used for analysis of *Zanthoxylum* species. In addition, some DNA sequences, including ITS nrDNA (Shen et al., 2005) and chloroplast regions (*matK*,* rbcL*,* trnL‐trnF*), have been used to assess the phylogeny of the *Zanthoxylum* genus (Feng et al., 2016b).

Herein, we investigated the utility and species identification ability of DNA barcoding using ITS2, ETS, and *trnH‐psbA* in Chinese *Zanthoxylum* and evaluated the ability to discriminate below the species level in this genus.

## MATERIALS AND METHODS

### Plant materials

A total of 69 individuals of 13 species were included in this study. Samples were from two sources: (1) 61 field collection specimens from all provinces in China presently known to contain *Zanthoxylum*, namely Shaanxi, Yunnan, Guizhou, Sichuan, Gansu, Hebei, Chongqing, and Shanxi, and (2) eight vouchered herbarium specimens from the Northwest A&F University Herbaria. Of the vouchered specimens, seven species were represented by more than one individual, and six species were represented by a single individual. These individuals represent a diverse mix of cultivated and wild *Zanthoxylum* species. Spatial coordinates were registered using a Global Positioning System (GPS) (Appendix S1).

### DNA extraction, amplification, and sequencing

Genomic DNA was extracted from either silica‐dried leaves or herbarium specimens using the modified cetyltrimethylammonium bromide procedure (Porebski et al., 1997). Universal primers for selected regions and sources are listed in Table 1. PCR amplification was conducted in 40‐μL volumes containing 20 μL of 2× *Taq* Master Mix (CWBIO Biotechnology Co. Ltd., Beijing, China), 2.0 μL of genomic DNA, 0.5 μL of each primer, and 17 μL of double‐distilled water. Cycling parameters for amplifying the ITS2 markers were as described previously by Chen et al. (2010). Cycling parameters for the ETS region were 96°C for 5 min; followed by 30 cycles of 96°C for 30 s, 55°C for 45 s, and 72°C for 40 s; and a final extension at 72°C for 5 min. Cycling parameters for the *trnH‐psbA* region were 94°C for 5 min; followed by 30 cycles of 94°C for 45 s, 57°C for 30 s, and 72°C for 72 s; and a final extension at 72°C for 10 min. PCR products were run on 1.5% agarose gels and sequenced by AuGCT DNA‐SYN Biotechnology (Beijing, China). All sequences reported in this study have been deposited in the National Center for Biotechnology Information (NCBI) GenBank database (accession numbers MF070103–MF070230, MF039484–MF039544; Appendix S2).

**Table 1:** Primers used for amplification of the ITS2, ETS, and *trnH‐psbA* regions of *Zanthoxylum* species

DNA barcoding region	Primer sequences (5′–3′)	Source
ITS2	F: ATGCGATACTTGCTGTGAAT	Chen et al., 2010
	R: GACGCTTCTCCAGACTACAAT	Chen et al., 2010
ETS	F: ATAGAGCGCGTGAGTGGTG	Mo, 2015
	R: GACAAGCATATGACTACTGGCAGGATCAA	Baldwin and Markos, 1998
*trnH‐psbA*	F: CGCGCATGGTGGATTCACAATCC	Tate and Simpson, 2003
	R: GTTATGCATGAACGTAATGCTC	Sang et al., 1997

### Data analysis

ITS2 sequences were annotated using ITS2 annotation tools based on the hidden Markov model to remove 5.8S and 28S DNA sequences (Keller et al., 2009). Sequences of three candidate regions were subsequently aligned with ClustalW, and genetic distances were calculated using MEGA 6.0 based on the Kimura 2‐parameter (K2P) model (Tamura et al., 2013). The three parameters calculated to characterize interspecific divergence using the K2P model were average interspecific distances, average theta prime (mean genetic variation between different species, thus eliminating biases associated with different numbers of samples among species), and smallest interspecific distances (the minimum interspecific distance within each genus was at least two species) (Meyer and Paulay, 2005; Chen et al., 2010; Gao et al., 2010). We also used the parameters average intraspecific distance, theta, and coalescent theta to evaluate the intraspecific distances based on the K2P model (Meyer and Paulay, 2005; Chen et al., 2010; Gao et al., 2010). The distribution of interspecific and intraspecific divergence was compared using DNA barcoding gaps (Moritz and Cicero, 2004; Chen et al., 2010). Wilcoxon signed‐rank tests were calculated using IBM SPSS Statistics version 19.0 (Meyer and Paulay, 2005).

We also applied BLAST and tree‐building methods to evaluate the species authentication capacity. In the BLAST method, all regions of *Zanthoxylum* species were used as query sequences. Correct identification was concluded when the best BLAST hit of the query sequence was from the expected species, ambiguous identification was concluded when the best BLAST hits for a query sequence were from several species including the expected species, and incorrect identification was concluded when the best BLAST hit was not from the expected species (Gao et al., 2010; van der Merwe et al., 2014). The tree‐building method was performed by constructing neighbor‐joining trees in MEGA 6.0 using the K2P model with bootstrap support values for individual clades computed by running 1000 bootstrap replicates. Species were considered identified if all individuals of a species formed a monophyletic group (Li et al., 2011). *Citrus limetta* Risso, *C. cavaleriei* H. Lév., and *Toddalia asiatica* (L.) Lam. were used as outgroups.

## RESULTS

### Amplification and sequencing of ITS2, ETS, and *trnH‐psbA*

In this study, two nuclear DNA regions and one chloroplast region were selected as candidate barcodes. Analysis of the efficiency of PCR amplification showed that ITS2 and *trnH‐psbA* achieved the highest rate (100%), followed by ETS (97.10%, except for the samples of *Z. echinocarpum* Hemsl. and *Z. multijugum* Franch.; Appendix S3). The sequencing success rates of ITS2 and ETS were 88.41% and 94.02%, respectively, while that of *trnH‐psbA* was 94.20% (Table 2). The aligned lengths of the ITS2 region ranged from 222 bp for *Z. piperitum* DC. to 227 bp for *Z. armatum*, and the GC content was highest for *Z. dissitum* Hemsl. (75.0%) and lowest for *Z. bungeanum* (66.6%). The aligned lengths of ETS sequences ranged from 439 bp for *Z. scandens* Blume to 447 bp for *Z. bungeanum* and *Z. armatum*; the GC content was highest for *Z. ailanthoides* Siebold & Zucc. (67.7%) and lowest for *Z. molle* Rehder (60.1%). The lengths of the *trnH‐psbA* plastid region extended from 430 bp (*Z. dissitum*) to 472 bp (*Z. piperitum*), and the GC content ranged from 29.2% (*Z. molle*) to 31.4% (*Z. multijugum*). The percentage of variable sites was lowest for *trnH‐psbA* (15.25%) and highest for ETS (27.29%; Table 3). Thus, the lengths and GC content of the three candidate regions for the tested species were relatively variable.

**Table 2:** Amplification and sequencing of the ITS2, ETS, and *trnH‐psbA* regions of *Zanthoxylum* species

DNA barcoding region	Amplification success	Amplification success rate (%)	Sequencing success	Sequencing success rate (%)	Amplification and sequencing success rate (%)
ITS2	69	100	61	88.41	88.41
ETS	67	97.10	63	94.02	91.30
*trnH‐psbA*	69	100	65	94.20	94.20

**Table 3:** Length, variation, and GC content of the ITS2, ETS, and *trnH‐psbA* regions of *Zanthoxylum* species

DNA barcoding region	Sequence length (bp)	Mean GC content (%)	No. of variable sites	Percentage of variable sites
ITS2	222–227	66.6–75.0	52	22.91
ETS	439–447	60.1–67.7	122	27.29
*trnH‐psbA*	430–472	29.5–30.8	72	15.25

### Assessment of intraspecific and interspecific genetic divergence

We used MEGA 6.0 based on the K2P model to estimate the genetic divergence of all species. The ITS2 and ETS regions displayed the highest interspecific and intraspecific divergence according to average interspecific distances, theta prime, average intraspecific distances, coalescent depth, and theta, while the *trnH‐psbA* region yielded lower values for these parameters (Table 4). Moreover, Wilcoxon signed‐rank tests confirmed that the ITS2 and ETS regions exhibited the highest interspecific variability and shared similar divergence, whereas variability and divergence were somewhat lower for the *trnH‐psbA* region (Table 5). For intraspecific divergence, Wilcoxon signed‐rank tests indicated that *trnH‐psbA* showed the lowest variation between conspecific individuals, whereas ETS showed the highest (Table 6).

**Table 4:** Analysis of interspecific divergence and intraspecific variation of the ITS2, ETS, and *trnH‐psbA* regions of *Zanthoxylum* species

Parameter	ITS2	ETS	*trnH‐psbA*
Interspecific distance	0.0798 ± 0.0338	0.0854 ± 0.0366	0.0454 ± 0.0269
Theta prime	0.0772 ± 0.0101	0.0880 ± 0.0141	0.0466 ± 0.0137
Minimum interspecific	0.0307 ± 0.0201	0.0380 ± 0.0276	0.0123 ± 0.0121
Intraspecific distance	0.0091 ± 0.0110	0.0103 ± 0.0102	0.0005 ± 0.0014
Theta	0.0046 ± 0.0042	0.0077 ± 0.0110	0.0003 ± 0.0008
Coalescent depth	0.0065 ± 0.0123	0.0117 ± 0.0128	0.0006 ± 0.0017

**Table 5:** Wilcoxon signed‐rank tests for interspecific divergence among the ITS2, ETS, and *trnH‐psbA* regions in *Zanthoxylum* species

W+	W−	Relative rank	*n*	*P* value	Result
ITS2	ETS	W+ = 608.5 W− = 931.5	55	≤0.176	ITS2 = ETS
ITS2	*trnH‐psbA*	W+ = 1318.5 W− = 221.5	55	≤4.31 × 10^−6^	ITS2 >> *trnH‐psbA*
ETS	*trnH‐psbA*	W+ = 1349 W− = 191	55	≤1.23 × 10^−6^	ETS >> *trnH‐psbA*

Note

*n* = sum of ranks.

**Table 6:** Wilcoxon signed‐rank tests for intraspecific divergence among the ITS2, ETS, and *trnH‐psbA* regions in *Zanthoxylum* species

W+	W−	Relative rank	*n*	*P* value	Result
ITS2	ETS	W+ = 31,711 W− = 39,165	453	≤0.077	ITS2 < ETS
ITS2	*trnH‐psbA*	W+ = 33,753 W− = 700	453	≤1.38 × 10^−41^	ITS2 >> *trnH‐psbA*
ETS	*trnH‐psbA*	W+ = 49,986 W− = 417	493	≤2.86 × 10^−52^	ETS >> *trnH‐psbA*

Note

*n* = sum of ranks.

### DNA barcoding gap and species discrimination

**Figure 1:**
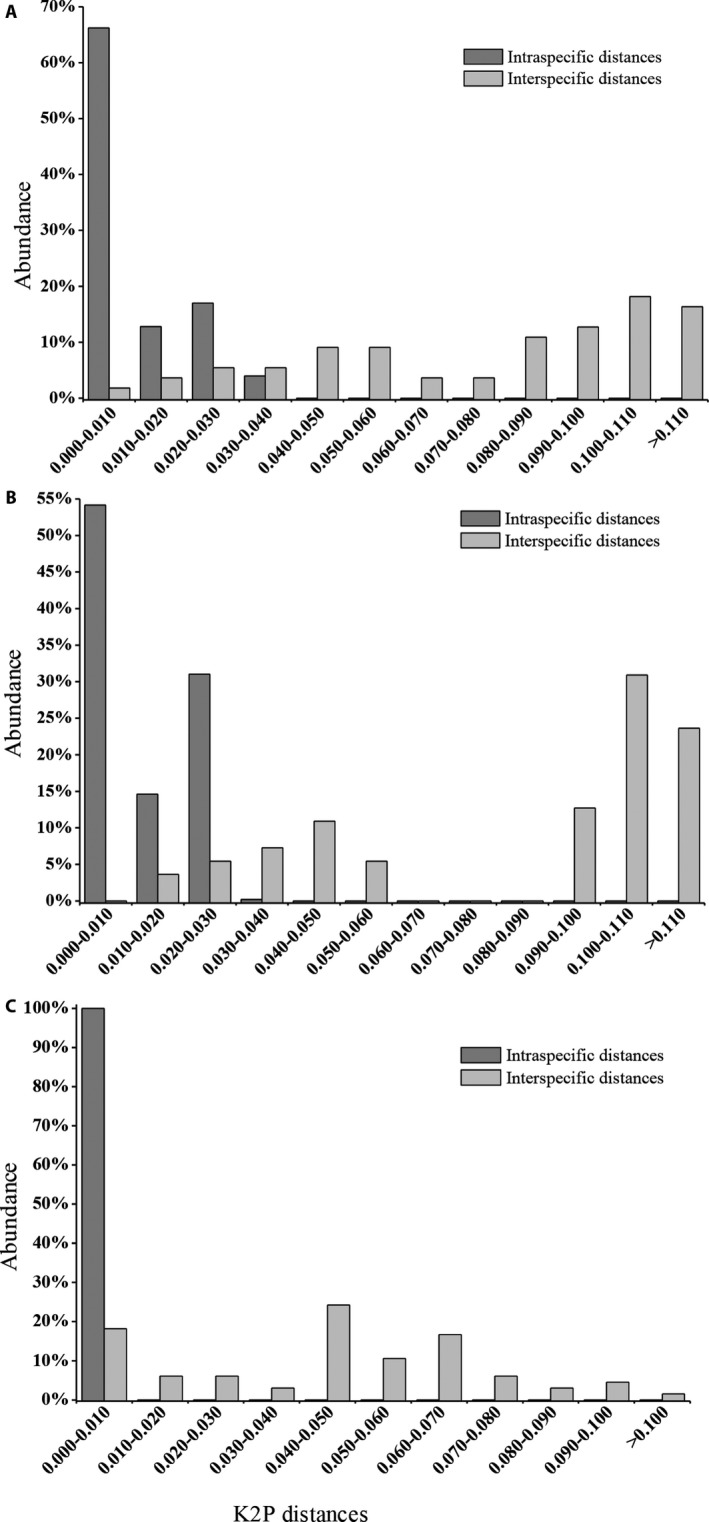
Relative distribution of interspecific divergence and intraspecific variation between *Zanthoxylum* species using the ITS2 (A), ETS (B), and *trnH‐psbA* (C) regions.

Based on the K2P model of intraspecific and interspecific divergence, we investigated the distribution of genetic distance among *Zanthoxylum* species at a scale of 0.01 distance units. The results indicated that none of the three regions exhibited distinct DNA barcoding gaps, and each distribution showed a slight overlap between intra‐ and interspecific distances (Fig. 1). In order to assess the identification efficiency of each region, we calculated the discrimination capacity using the BLAST and tree‐building methods. Regardless of whether the BLAST or the tree‐building method was used, the results showed that the ITS2 region had the highest discrimination efficacy (100%) at the species level. Meanwhile, ETS possessed 90.91% identification success rates at the species level for both the BLASTA1 method and the tree‐building method. The discrimination efficacy of the *trnH‐psbA* region is relatively lower (BLAST: 75.0%, tree‐building: 91.67%) (Table 7).

**Table 7:** Identification efficiency for the ITS2, ETS, and *trnH‐psbA* regions using the BLAST and tree‐building methods for species identification

DNA barcoding region	No. of samples	Correct identification rate, BLAST (%)	Correct identification rate, tree‐building (%)
ITS2	61	100	100
ETS	63	90.91	90.91
*trnH‐psbA*	65	75.0	91.67

### Phylogenetic analysis

**Figure 2:**
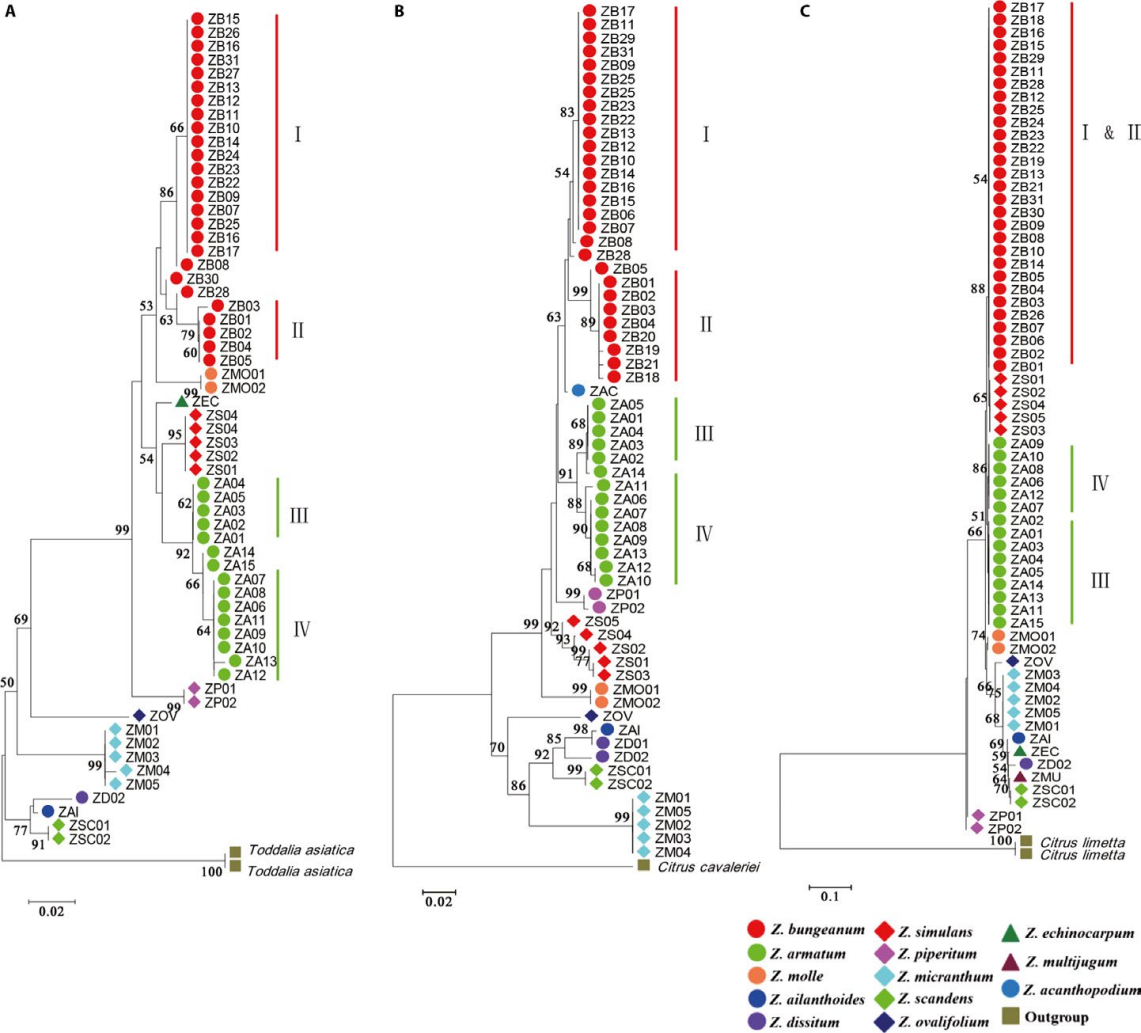
Neighbor‐joining trees based on ITS2 (A), ETS (B), and *trnH‐psbA* (C) sequences of *Zanthoxylum* species. Numbers above branches indicate bootstrap support (BS ≥ 50). Bar indicates 10% sequence variance. *Citrus limetta*,* C. cavaleriei*, and *Toddalia asiatica* were used as outgroups.

The primary purpose of the phylogenetic tree was not to evaluate the evolutionary relationships, but to test whether species are recovered as monophyletic, using phylogenetic techniques and bootstrap resampling. Our neighbor‐joining tree showed that the ITS2 region could distinguish between all tested species of the genus *Zanthoxylum* in 100% of cases; each species clustered as a distinct monophyletic group (Fig. 2A). However, two samples of *Z. dissitum* did not cluster together using the ETS region, and two samples of *Z. piperitum* did not cluster together using *trnH‐psbA* (Fig. 2B, C). Interestingly, we found that the *Z. armatum* accessions divided into two subgroups (III and IV) using all three regions. Subgroup III mainly consisted of cultivar accessions from ‘Dingtanhuajiao’ (ZA01–ZA05), ‘Jiangjinqinghuajiao’ (ZA14), and ‘Tengjiao’ (ZA15), whereas accessions of subgroup IV were wild varieties. In addition, the *Z*. *armatum* cultivar ‘Dingtanhuajiao’ is distributed exclusively in Guizhou Province and was recovered as monophyletic by ITS2 and ETS regions. Separation between cultivars in terms of phylogeny was also observed in *Z. bungeanum*, and ‘Hanchengdahongpao’ (ZB06–ZB09 and ZB22–ZB25) and ‘Fengxiandahongpao’ (ZB01–ZB05) were clearly clustered by ITS2 and ETS regions into subgroups I and II. Together, these findings indicate that the ITS2 region possesses powerful discriminatory ability both at and below the species level.

## DISCUSSION

A rapid and accurate method for authenticating species from the genus *Zanthoxylum* is important to ensure the safe use of drugs made from these medicinal herbs, as well as to maximize their economic value. DNA barcoding is a relatively new taxonomic method for fast, efficient, and reliable species identification (Hebert et al., 2004; Chen et al., 2010). An ideal DNA barcode should be routinely retrievable with a single universal primer pair and should exhibit high taxonomic coverage and high resolution. Given all these considerations, we tested three regions (ITS2, ETS, and *trnH‐psbA*) for their utility as DNA barcode targets to distinguish 13 *Zanthoxylum* species. To the best of our knowledge, this is the first study using these three regions for species identification in the *Zanthoxylum* genus.

Compared with the *trnH‐psbA* barcode in the chloroplast region, the ITS2 and ETS regions showed higher interspecific variation and discriminatory power, and accurately discriminated all *Zanthoxylum* species tested in this study. Specifically, ITS2 distinguished 100% of monophyletic species, and performed better than the other two loci. The nuclear ITS2 region is often regarded as a candidate DNA barcode because of its valuable characteristics, including availability of conserved regions for designing universal primers, ease of amplification, and sufficient variability to distinguish even closely related species (Chen et al., 2010).

The ETS nuclear region has previously been used as a DNA barcode for the genus *Paulownia* (Mo, 2015). However, our current results indicate that ETS is not an ideal candidate DNA barcode for the *Zanthoxylum* genus, based on the criteria applied. First, the length of ITS2 is relatively short compared to ETS. Second, the PCR amplification efficiency of ITS2 was higher than that of ETS. Third, using the BLAST method, ITS2 achieved a higher success rate for identification of samples at the species level compared with ETS. Although ETS had higher interspecific divergence than ITS2 in our study, Wilcoxon signed‐rank tests indicated no statistical differences between the two regions (Tables 5, 6).

In addition, the phylogenetic tree indicated that the ITS2 and ETS regions showed powerful discriminatory ability not only at the species level, but also below the species level. The Qingling Mountains form the boundary between southern and northern China; the ‘Hanchengdahongpao’ accession is perhaps the most well‐known representative of *Z. bungeanum* in northern China, whereas ‘Fengxiandahongpao’ is the most common variety in the south. Based on our phylogenetic analysis, these representative accessions were identified correctly using both ITS2 and ETS regions, and the ‘Dingtanhuajiao’ cultivated accession of *Z. armatum* could also be distinguished from the other cultivated accessions by both ITS2 and ETS (Fig. 2A, B). These results imply that ITS2 is superior to ETS as a DNA marker in the *Zanthoxylum* genus and that the ETS spacer should be used as a complementary barcode in the *Zanthoxylum* genus.

In previous studies, the chloroplast region *trnH‐psbA* was dismissed early on because of considerable interspecific variation, and even intraspecific variation, including the presence of inversions and insertion‐deletion polymorphisms (indels), which make it difficult to align among broad groups of taxa (Fazekas et al., 2008; Whitlock et al., 2010). In the present study, we did not find these problems with *Zanthoxylum*. However, using the full data set constructed in this study, the *trnH‐psbA* region proved unsuitable for DNA barcoding in *Zanthoxylum*. For example, using the BLAST method, *trnH‐psbA* had the lowest correct identification rate at the species level among the three regions tested. Moreover, this limitation is supported by the wide variation in sequence length. Variable lengths complicate sequence alignment, which increases the difficulty and lowers the accuracy in real‐world applications. In addition, the *trnH‐psbA* region exhibited lower interspecific and intraspecific divergence than the ITS2 and ETS regions. All of these results indicate that the *trnH‐psbA* region does not have potential for DNA barcoding in Chinese *Zanthoxylum*.

Surprisingly, herbarium materials accounted for most of the failed amplification and sequencing results, and removing these results increased the sequencing success rate for ITS2, ETS, and *trnH‐psbA* from 88.41%, 94.02%, and 94.20% to 94.20%, 95.52%, and 98.55%, respectively. We believe this may be related to primer design or fundamental changes in gene structure during herbarium specimen preparation and storage, but it could also be related to the existence of homologous sequences (Starr et al., 2009; Xiao et al., 2010; Hollingsworth et al., 2011). Thus, sequencing technology should be improved to obtain more high‐quality sequences. In addition, Kress et al. (2005) advocated that using well‐preserved specimens is important for successful DNA barcoding. However, herbarium specimens often vary in how and when they are dried after being pressed, and it is not always certain that specimens were stored in a good state. We collected more than one herbarium sample for each species, but DNA could not be extracted from some samples, and six species included only a single individual. Therefore, we agree that it is best to use fresh samples for DNA barcoding.

Ideally, a perfect DNA barcode should have a distinct DNA barcoding gap and no overlap (Meyer and Paulay, 2005). However, none of the three loci in this study have a distinct DNA barcoding gap (Fig. 1). Moritz and Cicero (2004) suggested that the overlap is considerably greater when a larger proportion of closely related taxa are included. Our results suggest that regions in some cultivars (*Z*. *bungeanum* and *Z*. *armatum*) have undergone strong natural or artificial selection. A previous study indicated that cultivated *Zanthoxylum* species experienced multiple long‐distance dispersal events, as well as several vicariance events, and the ancestors of *Zanthoxylum* first colonized the Chinese provinces of Yunnan and Guizhou (Feng et al., 2016b). Our *Zanthoxylum* samples included both wild and cultivated varieties, which led to maximum intraspecific distances that were greater than minimum interspecific distances.

The essence of DNA barcoding technology is the use of a universal barcode. However, in reality, and regardless of the DNA barcoding employed, there will always be some species that would be better resolved by a specific DNA barcode (Pang et al., 2012). Numerous studies have focused on a large number of plant species with distant genetic relationships, but the present study is one of only a few to evaluate the efficacy of DNA barcoding among congeneric *Zanthoxylum* species. In recent years, various studies have proved that ITS2 is a universal barcode in Asteraceae, Fabaceae, and other families (Gao et al., 2010; Liu et al., 2010). Chen et al. (2010) compared seven candidate DNA barcoding regions (ITS2, *matK*,* rbcL*,* trnH‐psbA*,* ycf5*, and *rpoC1*) and proposed that ITS2 can be used as a standard DNA barcode in medicinal plants. As far as we know, DNA barcoding is currently used for identification at the species level, and there are few reports of identification at cultivar level. According to the data of ITS2 sequence characteristics, intra‐ and interspecific K2P genetic distances, and a neighbor‐joining algorithm, relatively large variation occurred not only at the species level but also at the cultivar level. For example, the sequence characteristics also revealed that the cultivars ‘Hangchengdahong,’ ‘Fengxiandahongpao’ (*Z. bungeanum*), and ‘Dingtanhuajiao’ (*Z. armatum*) exhibit more divergence than other cultivars, probably resulting from their wide distribution and diverse habitats. The variation of nuclear ribosomal ITS2 sequences within Chinese *Zanthoxylum* cultivars may stem from the domestication process and the geographical barrier. The two *Z. bungeanum* cultivars are distributed south and north of the Qinling Mountains, which block the genetic flow of the two cultivars and create a unique genetic divergence. In our study, the ITS2 data demonstrate 100% accuracy in identifying the Chinese *Zanthoxylum* species using both the BLAST and tree‐building methods. Furthermore, the ITS2 sequence length is short in *Zanthoxylum*, which is suitable for the PCR amplification and sequencing. We believe that, in addition to its use as a standard phylogenetic marker, ITS2 is a useful DNA barcode in Chinese *Zanthoxylum* species. We therefore recommend the nuclear ITS2 region for DNA barcoding in Chinese *Zanthoxylum* and its application in Chinese *Zanthoxylum* germplasm collections and breeding programs. Our results provide valuable baseline data for the development of more efficient conservation and management plans for this important aromatic species.

## DATA ACCESSIBILITY

The raw data of three DNA barcoding sequences of 69 *Zanthoxylum* accessions have been deposited in GenBank (accession no. MF070103–MF070230 and MF039484–MF039544).

## Supporting information

Appendix S1

Appendix S2

Appendix S3

## References

[aps31157-bib-0001] Acevedo‐Rosas, R. , K. Cameron , and V. Sosa . 2004 A molecular phylogenetic study of *Graptopetalum* (Crassulaceae) based on ETS, ITS, *rpl16*, and *trnL‐F* nucleotide sequences. American Journal of Botany 91: 1099–1104. 21653465 10.3732/ajb.91.7.1099

[aps31157-bib-0002] Baldwin, B. G. , and S. Markos . 1998 Phylogenetic utility of the external transcribed spacer (ETS) of 18S‐26S rDNA: Congruence of ETS and ITS trees of *Calycadenia* (Compositae). Molecular Phylogenetics and Evolution 10: 449–463. 10051397 10.1006/mpev.1998.0545

[aps31157-bib-0003] Bryant, B. P. , and I. Mezine . 1999 Alkylamides that produce tingling paresthesia activate tactile and thermal trigeminal neurons. Brain Research 842: 452–460. 10526142 10.1016/s0006-8993(99)01878-8

[aps31157-bib-0004] CBOL Plant Working Group . 2009 A DNA barcode for land plants. Proceedings of the National Academy of Sciences USA 106: 12794–12797. 10.1073/pnas.0905845106 PMC2722355 19666622

[aps31157-bib-0005] Chase, W. , N. Salamin , M. Wilkinson , J. M. Dunwell , P. K. Rao , N. Haidar , and V. Savolainen . 2005 Land plants and DNA barcodes: Short‐term and long‐term goals. Philosophical Transactions of the Royal Society of London 360: 1889. 16214746 10.1098/rstb.2005.1720 PMC1609218

[aps31157-bib-0006] Chen, S. , H. Yao , J. Han , C. Liu , J. Song , L. Shi , and Y. Zhu . 2010 Validation of the ITS2 region as a novel DNA barcode for identifying medicinal plant species. PLoS One 5: e8613. 20062805 10.1371/journal.pone.0008613 PMC2799520

[aps31157-bib-0007] Cheng, J. , X. Lee , B. K. G. Theng , L. Zhang , B. Fang , and F. Li . 2015 Biomass accumulation and carbon sequestration in an age‐sequence of *Zanthoxylum bungeanum* plantations under the Grain for Green Program in karst regions, Guizhou province. Agricultural and Forest Meteorology 203: 88–95.

[aps31157-bib-0008] Cowan, R. S. , M. W. Chase , W. J. Kress , and V. Savolainen . 2006 300,000 Species to identify: Problems, progress, and prospects in DNA barcoding of land plants. Taxon 55: 611–616.

[aps31157-bib-0009] Erickson, D. L. , J. Spouge , A. Resch , L. A. Weight , and W. J. Kress . 2008 DNA barcoding in land plants: Developing standards to quantify and maximize success. Taxon 57: 1304–1316. 19779570 PMC2749701

[aps31157-bib-0010] Fazekas, A. J. , K. S. Burgess , P. R. Kesanakurti , S. W. Graham , S. G. Newmaster , B. C. Husband , D. M. Percy , et al. 2008 Multiple multilocus DNA barcodes from the plastid genome discriminate plant species equally well. PLoS One 3(7): e2802. 18665273 10.1371/journal.pone.0002802 PMC2475660

[aps31157-bib-0011] Feng, S. , T. Yang , Z. Liu , L. Chen , N. Hou , Y. Wang , and A. Wei . 2015 Genetic diversity and relationships of wild and cultivated *Zanthoxylum* germplasms based on sequence‐related amplified polymorphism (SRAP) markers. Genetic Resources and Crop Evolution 62: 1193–1204.

[aps31157-bib-0012] Feng, S. , M. Jiang , Y. Shi , K. Jiao , C. Shen , J. Lu , Q. Ying , and H. Wang . 2016a Application of the ribosomal DNA ITS2 region of *Physalis* (Solanaceae): DNA barcoding and phylogenetic study. Frontiers in Plant Science 7: 1047. 27486467 10.3389/fpls.2016.01047 PMC4949264

[aps31157-bib-0013] Feng, S. , Z. Liu , L. Chen , N. Hou , T. Yang , and A. Wei . 2016b Phylogenetic relationships among cultivated *Zanthoxylum* species in China based on cpDNA markers. Tree Genetics and Genomes 12: 45.

[aps31157-bib-0014] Gao, T. , H. Yao , J. Song , Y. Zhu , X. Ma , X. Pang , H. Xu , and S. Chen . 2010 Identification of medicinal plants in the family Fabaceae using a potential DNA barcode ITS2. Journal of Ethnopharmacology 130: 116–121. 20435122 10.1016/j.jep.2010.04.026

[aps31157-bib-0015] Han, J. , X. Pang , B. Liao , H. Yao , J. Song , and S. Chen . 2016 An authenticity survey of herbal medicines from markets in China using DNA barcoding. Scientific Reports 6: 18723. 26740340 10.1038/srep18723 PMC4703975

[aps31157-bib-0016] Hebert, P. D. , A. Cywinska , S. L. Ball , and J. R. Dewaard . 2003 Biological identifications through DNA barcodes. Proceedings of the Royal Society, B, Biological Sciences 270: 313. 12614582 10.1098/rspb.2002.2218 PMC1691236

[aps31157-bib-0017] Hebert, P. D. , E. H. Penton , J. M. Burns , D. H. Janzen , and W. Hallwaches . 2004 Ten species in one: DNA barcoding reveals cryptic species in the neotropical skipper butterfly *Astraptes fulgerator* . Proceedings of the National Academy of Sciences USA 101: 14812. 10.1073/pnas.0406166101 PMC522015 15465915

[aps31157-bib-0018] Hollingsworth, P. M. , S. W. Graham , and D. P. Little . 2011 Choosing and using a plant DNA barcode. PLoS One 6: e19254. 21637336 10.1371/journal.pone.0019254 PMC3102656

[aps31157-bib-0019] Keller, A. , T. Schleicher , J. Schultz , T. Müller , T. Dandekar , and M. Wolf . 2009 5.8S‐28S rRNA interaction and HMM‐based ITS2 annotation. Gene 430: 50–57. 19026726 10.1016/j.gene.2008.10.012

[aps31157-bib-0020] Kool, A. , H. J. De Boer , A. Krüger , A. Rydberg , A. Abbad , L. Bjorkörk , and G. Martin . 2012 Molecular identification of commercialized medicinal plants in southern Morocco. PLoS One 7: e39459. 22761800 10.1371/journal.pone.0039459 PMC3384669

[aps31157-bib-0021] Kress, W. J. , K. J. Wurdack , E. A. Zimmer , L. A. Weigt , and D. H. Janzen . 2005 Use of DNA barcodes to identify flowering plants. Proceedings of the National Academy of Sciences USA 102: 8369. 10.1073/pnas.0503123102 PMC1142120 15928076

[aps31157-bib-0022] Lahaye, R. , M. van der Bank , D. Bogarin , J. Warner , F. Pupulin , G. Gigot , O. Maurin , et al. 2008 DNA barcoding the floras of biodiversity hotspots. Proceedings of the National Academy of Sciences USA 105: 2923. 10.1073/pnas.0709936105 PMC2268561 18258745

[aps31157-bib-0023] Li, D. , L. Gao , H. Li , H. Wang , X. Ge , J. Liu , Z. Chen , et al. 2011 Comparative analysis of a large dataset indicates that internal transcribed spacer (ITS) should be incorporated into the core barcode for seed plants. Proceedings of the National Academy of Sciences USA 108: 19641–19646. 10.1073/pnas.1104551108 PMC3241788 22100737

[aps31157-bib-0024] Li, J. , J. H. Alexander , and D. Zhang . 2002 Paraphyletic *Syringa* (Oleaceae): Evidence from sequences of nuclear ribosomal DNA ITS and ETS regions. Systematic Botany 27: 592–597.

[aps31157-bib-0025] Li, L. , J. Li , J. G. Conran , and X. W. Li . 2007 Phylogeny of *Neolitsea* (Lauraceae) inferred from Bayesian analysis of nrDNA ITS and ETS sequences. Plant Systematics and Evolution 269: 203–221.

[aps31157-bib-0026] Li, X. , L. Yue , C. Xie , X. Li , Y. Yu , Y. Meng , and S. Chen . 2016 The chemical and genetic characteristics of Szechuan pepper (*Zanthoxylum bungeanum* and *Z. armatum*) cultivars and their suitable habitat. Frontiers in Plant Science 7: https://doi.org/10.3389/fpls.2016.00467. 10.3389/fpls.2016.00467 PMC4835500 27148298

[aps31157-bib-0027] Liu, C. , Y. Zhu , J. Song , H. Yao , T. Gao , and S. Chen . 2010 Evaluating the feasibility of using candidate DNA barcodes in discriminating species of the large Asteraceae family. BMC Evolutionary Biology 10: 324. 20977734 10.1186/1471-2148-10-324 PMC3087544

[aps31157-bib-0028] Meyer, C. P. , and G. Paulay . 2005 DNA barcoding: Error rates based on comprehensive sampling. PLoS Biology 3: e422. 16336051 10.1371/journal.pbio.0030422 PMC1287506

[aps31157-bib-0029] Mo, W. 2015 Evaluating the feasibility of candidate DNA barcodes for genus Paulownia. Chinese Academy of Forestry, Beijing, China.

[aps31157-bib-0030] Moritz, C. , and C. Cicero . 2004 DNA barcoding: Promise and pitfalls. PLoS Biology 2: e354. 15486587 10.1371/journal.pbio.0020354 PMC519004

[aps31157-bib-0031] Newmaster, S. G. , A. J. Fazekas , R. A. D. Steeves , and J. Janovec . 2008 Testing candidate plant barcode regions in the Myristicaceae. Molecular Ecology Resources 8: 480–490. 21585825 10.1111/j.1471-8286.2007.02002.x

[aps31157-bib-0032] Pang, X. , L. Chang , L. Shi , L. Rui , L. Dong , H. Li , S. S. Cherny , and S. Chen . 2012 Utility of the *trnH‐psbA* intergenic spacer region and its combinations as plant DNA barcodes: A meta‐analysis. PLoS One 7: e48833. 23155412 10.1371/journal.pone.0048833 PMC3498263

[aps31157-bib-0033] Patiño, L. O. J. , R. J. A. Prieto , and S. L. E. Cuca . 2008 *Zanthoxylum* genus as potential source of bioactive compounds *In* Rasooli I. [ed.], Bioactive compounds in phytomedicine, 185–218. InTech China, Shanghai, China.

[aps31157-bib-0034] Pennisi, E. 2007 Wanted: A barcode for plants. Science 318: 190–191. 17932267 10.1126/science.318.5848.190

[aps31157-bib-0035] Piredda, R. , M. C. Simeone , M. Attimonelli , R. Bellarosa , and B. Schirone . 2011 Prospects of barcoding the Italian wild dendroflora: Oaks reveal severe limitations to tracking species identity. Molecular Ecology Resources 11: 72–83. 21429102 10.1111/j.1755-0998.2010.02900.x

[aps31157-bib-0036] Porebski, S. , L. G. Bailey , and B. R. Baum . 1997 Modification of a CTAB DNA extraction protocol for plants containing high polysaccharide and polyphenol components. Plant Molecular Biology Reporter 15: 8–15.

[aps31157-bib-0037] Sang, T. , D. Crawford , and T. Stuessy . 1997 Chloroplast DNA phylogeny, reticulate evolution, and biogeography of *Paeonia* (Paeoniaceae). American Journal of Botany 84: 1120–1136. 21708667

[aps31157-bib-0038] Sass, C. , D. P. Little , D. W. Stevenson , and C. D. Specht . 2007 DNA barcoding in the Cycadales: Testing the potential of proposed barcoding markers for species identification of cycads. PLoS One 2: e1154. 17987130 10.1371/journal.pone.0001154 PMC2063462

[aps31157-bib-0039] Schultz, J. , S. Maisel , D. Gerlach , T. Müller , and M. Wolf . 2005 A common core of secondary structure of the internal transcribed spacer 2 (ITS2) throughout the Eukaryota. RNA 11: 361–364. 15769870 10.1261/rna.7204505 PMC1370725

[aps31157-bib-0040] Shen, J. , X. Ding , W. Zhang , S. Bao , J. Chang , and F. Tang . 2005 Authentication of *Zanthoxylum bungeanum* Maxin population and adulterants by analysis of rDNA ITS sequences. Acta Pharmaceutica Sinica 40: 80–86. 15881332

[aps31157-bib-0041] Starr, J. R. , R. F. C. Naczi , and B. N. Chouinard . 2009 Plant DNA barcodes and species resolution in sedges (*Carex*, Cyperaceae). Molecular Ecology Resources 9: 151–163. 21564974 10.1111/j.1755-0998.2009.02640.x

[aps31157-bib-0042] Su, G. Y. , K. Wang , X. Wang , and B. Wu . 2015 Bioactive lignans from *Zanthoxylum planispinum* with cytotoxic potential. Phytochemistry Letters 11: 120–126.

[aps31157-bib-0043] Tamura, K. , G. Stecher , D. Peterson , A. Filipski , and S. Kumar . 2013 MEGA6: Molecular Evolutionary Genetics Analysis version 6.0. Molecular Biology and Evolution 30: 2725. 24132122 10.1093/molbev/mst197 PMC3840312

[aps31157-bib-0044] Tate, J. A. , and B. B. Simpson . 2003 Paraphyly of *Tarasa* (Malvaceae) and diverse origins of the polyploid species. Systematic Botany 28: 723–737.

[aps31157-bib-0045] Tu, Y. , C. Wei , and Z. Zuo . 2001 A new *Zanthoxylum* genus—*Z. planipinum* var. *dingtanensis* and the research of its species classification. Guizhou Science 19: 77–80.

[aps31157-bib-0046] van der Merwe, M. , H. McPherson , J. Siow , and M. Rossetto . 2014 Next‐gen phylogeography of rainforest trees: Exploring landscape‐level cpDNA variation from whole‐genome sequencing. Molecular Ecology Resources 14: 199–208. 24119022 10.1111/1755-0998.12176

[aps31157-bib-0047] Wei, A. , T. Yang , and L. Zhou . 2012 Guidelines for the production of Zanthoxylum safety. China Agricultural Press, Beijing, China.

[aps31157-bib-0048] Whitlock, B. A. , A. M. Hale , and P. A. Groff . 2010 Groff P: Intraspecific inversions pose a challenge for the *trnH‐psbA* plant DNA barcode. PLoS One 5(7): e11533. 20644717 10.1371/journal.pone.0011533 PMC2903610

[aps31157-bib-0049] Xiao, L. Q. , M. Möller , and H. Zhu . 2010 High nrDNA ITS polymorphism in the ancient extant seed plant *Cycas*: Incomplete concerted evolution and the origin of pseudogenes. Molecular Phylogenetics and Evolution 55: 168–177. 19945537 10.1016/j.ympev.2009.11.020

[aps31157-bib-0050] Yang, X. 2008 Aroma constituents and alkylamides of red and green huajiao (*Zanthoxylum bungeanum* and *Zanthoxylum schinifolium*). Journal of Agricultural and Food Chemistry 56: 1689. 18271544 10.1021/jf0728101

