# Supplementary material for: Internal transcribed spacer 2 (ITS2) barcodes: A useful tool for identifying Chinese Zanthoxylum
Source: Appl Plant Sci. 2018 Jun 15;6(6):e01157. doi: 10.1002/aps3.1157 (PMC6025816; doi:10.1002/aps3.1157)
Supplement: Supplementary file 1 — Appendix S1 [file APS3-6-e01157-s001.docx]

APPENDIX S1. Samples of Zanthoxylum species used in the present study.

| **Code** | **Sample type** | **Cultivar name** | **Species** | **Voucher**  **no.** | **Longitude** | **Latitude** | **Locality** |
| --- | --- | --- | --- | --- | --- | --- | --- |
| ZB01 | Cultivar | Fengxiandahongpao | *Z. bungeanum* | — | 106.51 | 33.91 | Fengxian Shaanxi |
| ZB02 | Cultivar | Fengxiandahongpao | *Z. bungeanum* | — | 106.51 | 33.91 | Fengxian Shaanxi |
| ZB03 | Cultivar | Fengxiandahongpao | *Z. bungeanum* | — | 106.51 | 33.91 | Fengxian Shaanxi |
| ZB04 | Cultivar | Fengxiandahongpao | *Z. bungeanum* | — | 106.51 | 33.91 | Fengxian Shaanxi |
| ZB05 | Cultivar | Fengxiandahongpao | *Z. bungeanum* | — | 106.51 | 33.91 | Fengxian Shaanxi |
| ZB06 | Cultivar | Hanchengdahongpao | *Z. bungeanum* | — | 110.27 | 35.44 | Hancheng Shaanxi |
| ZB07 | Cultivar | Hanchengdahongpao | *Z. bungeanum* | — | 110.27 | 35.44 | Hancheng Shaanxi |
| ZB08 | Cultivar | Hanchengdahongpao | *Z. bungeanum* | — | 110.27 | 35.44 | Hancheng Shaanxi |
| ZB09 | Cultivar | Hanchengdahongpao | *Z. bungeanum* | — | 110.27 | 35.44 | Hancheng Shaanxi |
| ZB10 | Cultivar | Hanyuanhuajiao | *Z. bungeanum* | — | 102.46 | 29.66 | Yaan Sichuan |
| ZB11 | Cultivar | Hanyuanhuajiao | *Z. bungeanum* | — | 102.46 | 29.66 | Yaan Sichuan |
| ZB12 | Cultivar | Hanyuanhuajiao | *Z. bungeanum* | — | 102.46 | 29.66 | Yaan Sichuan |
| ZB13 | Cultivar | Hanyuanhuajiao | *Z. bungeanum* | — | 102.46 | 29.66 | Yaan Sichuan |
| ZB14 | Cultivar | Hanyuanhuajiao | *Z. bungeanum* | — | 102.46 | 29.66 | Yaan Sichuan |
| ZB15 | Cultivar | Yunnandahongpao | *Z. bungeanum* | — | 105.66 | 25.68 | Kunming Yunnan |
| ZB16 | Cultivar | Yunnandahongpao | *Z. bungeanum* | — | 105.66 | 25.68 | Kunming Yunnan |
| ZB17 | Cultivar | Yunnandahongpao | *Z. bungeanum* | — | 105.66 | 25.68 | Kunming Yunnan |
| ZB18 | Cultivar | Qin’anyihao | *Z. bungeanum* | — | 105.63 | 35.05 | Qin'an Gansu |
| ZB19 | Cultivar | Qin’anyihao | *Z. bungeanum* | — | 105.63 | 35.05 | Qin'an Gansu |
| ZB20 | Cultivar | Qin’anyihao | *Z. bungeanum* | — | 105.63 | 35.05 | Qin'an Gansu |
| ZB21 | Cultivar | Qin’anyihao | *Z. bungeanum* | — | 105.63 | 35.05 | Qin'an Gansu |
| ZB22 | Cultivar | Hanchengdahongpao | *Z. bungeanum* | — | 110.27 | 35.44 | Hancheng Shanxi |
| ZB23 | Cultivar | Hanchengdahongpao | *Z. bungeanum* | — | 110.27 | 35.44 | Hancheng Shanxi |
| ZB24 | Cultivar | Hanchengdahongpao | *Z. bungeanum* | — | 110.27 | 35.44 | Hancheng Shanxi |
| ZB25 | Cultivar | Hanchengdahongpao | *Z. bungeanum* | — | 110.27 | 35.44 | Hancheng Shanxi |
| ZB26 | Cultivar | Gongjiao | *Z. bungeanum* | — | 102.46 | 29.65 | Hanyuan Sichuan |
| ZB27 | Herbarium |  | *Z. bungeanum* | WUK518576 | — | — | Northwest A&F  University Herbaria |
| ZB28 | Cultivar | Shanxihuajiao | *Z. bungeanum* | — | 110.68 | 34.70 | Ruicheng Shanxi |
| ZB29 | Cultivar | Gongjiao | *Z. bungeanum* | — | 103.48 | 29.60 | Emei Sichuan |
| ZB30 | Cultivar | Hebeihuajiao | *Z. bungeanum* | — | 113.67 | 36.57 | Shexian Hebei |
| ZB31 | Cultivar | Yunnanhuajiao | *Z. bungeanum* | — | 103.72 | 27.33 | Zhaotong Yunnan |
| ZA01 | Cultivar | Dingtanhuajiao | *Z. armatum* | — | 104.89 | 25.90 | Anshun Guizhou |
| ZA02 | Cultivar | Dingtanhuajiao | *Z. armatum* | — | 104.89 | 25.90 | Anshun Guizhou |
| ZA03 | Cultivar | Dingtanhuajiao | *Z. armatum* | — | 104.89 | 25.90 | Anshun Guizhou |
| ZA04 | Cultivar | Dingtanhuajiao | *Z. armatum* | — | 104.89 | 25.90 | Anshun Guizhou |
| ZA05 | Cultivar | Dingtanhuajiao | *Z. armatum* | — | 104.89 | 25.90 | Anshun Guizhou |
| ZA06 | Cultivar | Zhuyehuajiao | *Z. armatum* | — | 105.66 | 25.68 | Liupanshui Guizhou |
| ZA07 | Wild | Zhuyehuajiao | *Z. armatum* | — | 105.66 | 25.68 | Liupanshui Guizhou |
| ZA08 | Wild | Zhuyehuajiao | *Z. armatum* | — | 105.66 | 25.68 | Liupanshui Guizhou |
| ZA09 | Wild | Zhuyehuajiao | *Z. armatum* | — | 104.89 | 25.90 | Anshun Guizhou |
| ZA10 | Wild | Zhuyehuajiao | *Z. armatum* | — | 104.89 | 25.90 | Anshun Guizhou |
| ZA11 | Wild | Zhuyehuajiao | *Z. armatum* | — | 104.89 | 25.90 | Anshun Guizhou |
| ZA12 | Wild | Zhuyehuajiao | *Z. armatum* | — | 104.89 | 25.90 | Anshun Guizhou |
| ZA13 | Herbarium |  | *Z. armatum* | WUK0498189 | — | — | Northwest A&F  University Herbaria |
| ZA14 | Cultivar | Jiangjinqinghuajiao | *Z. armatum* | — | 106.16 | 29.18 | Chongqing |
| ZA15 | Cultivar | Tengjiao | *Z. armatum* | — | 103.48 | 29.60 | Emei Sichuan |
| ZP01 | Cultivar | Chaocanghuajiao | *Z. piperitum* | — | 105.05 | 29.58 | Neijiang Sichuan |
| ZP02 | Cultivar | Chaocanghuajiao | *Z. piperitum* | — | 105.05 | 29.58 | Neijiang Sichuan |
| ZS01 | Wild |  | *Z. simulans* | — | 106.73 | 33.95 | Fengxian Shaanxi |
| ZS02 | Wild |  | *Z. simulans* | — | 106.73 | 33.95 | Fengxian Shaanxi |
| ZS03 | Wild |  | *Z. simulans* | — | 106.73 | 33.95 | Fengxian Shaanxi |
| ZS04 | Wild |  | *Z. simulans* | — | 106.73 | 33.95 | Fengxian Shaanxi |
| ZS05 | Wild |  | *Z. simulans* | — | 106.73 | 33.95 | Fengxian Shaanxi |
| ZM01 | Wild |  | *Z. micranthum* | — | 104.89 | 25.90 | Liupanshui Guizhou |
| ZM02 | Wild |  | *Z. micranthum* | — | 104.89 | 25.90 | Liupanshui Guizhou |
| ZM03 | Wild |  | *Z. micranthum* | — | 104.89 | 25.90 | Liupanshui Guizhou |
| ZM04 | Wild |  | *Z. micranthum* | — | 104.89 | 25.90 | Liupanshui Guizhou |
| ZM05 | Wild |  | *Z. micranthum* | — | 104.89 | 25.90 | Liupanshui Guizhou |
| ZSC01 | Wild |  | *Z. scandens* | — | 104.89 | 25.90 | Liupanshui Guizhou |
| ZSC02 | Wild |  | *Z. scandens* | — | 104.89 | 25.90 | Liupanshui Guizhou |
| ZMO1 | Wild |  | *Z. molle* | — | 102.45 | 25.15 | Kunming Yunnan |
| ZMO2 | Wild |  | *Z. molle* | — | 102.45 | 25.15 | Kunming Yunnan |
| ZAI | Wild |  | *Z. ailanthoides* | — | 102.45 | 25.15 | Kunming Yunnan |
| ZAC | Herbarium |  | *Z. acanthopodium* | WUK0275511 | — | — | Northwest A&F  University Herbaria |
| ZEC | Herbarium |  | *Z. echinocarpum* | WUK0199918 | — | — | Northwest A&F  University Herbaria |
| ZMU | Herbarium |  | *Z. multijugum* | WUK0204157 | — | — | Northwest A&F  University Herbaria |
| ZD01 | Herbarium |  | *Z. dissitum* | WUK0206719 | — | — | Northwest A&F  University Herbaria |
| ZD02 | Herbarium |  | *Z. dissitum* | WUK0238215 | — | — | Guizhou Academy of Forestry |
| ZOV | Herbarium |  | *Z. ovalifolium* | WUK0495035 | — | — | Northwest A&F  University Herbaria |
