# Supplementary material for: Internal transcribed spacer 2 (ITS2) barcodes: A useful tool for identifying Chinese Zanthoxylum
Source: Appl Plant Sci. 2018 Jun 15;6(6):e01157. doi: 10.1002/aps3.1157 (PMC6025816; doi:10.1002/aps3.1157)
Supplement: Supplementary file 2 — Appendix S2 [file APS3-6-e01157-s002.docx]

APPENDIX S2. GenBank accession numbers of the ITS2, ETS, and *trnH-psbA* regions of *Zanthoxylum* species.

| **Code** | **Species** | **ITS2** | **ETS** | ***trnH-psbA*** |
| --- | --- | --- | --- | --- |
| ZB01 | *Z. bungeanum* | MF039487 | MF070109 | MF070171 |
| ZB02 | *Z. bungeanum* | MF039488 | MF070110 | MF070172 |
| ZB03 | *Z. bungeanum* | MF039489 | MF070121 | MF070198 |
| ZB04 | *Z. bungeanum* | MF039490 | MF070122 | MF070199 |
| ZB05 | *Z. bungeanum* | MF039490 | MF070123 | MF070200 |
| ZB06 | *Z. bungeanum* | MF039492 | MF070173 | MF070173 |
| ZB07 | *Z. bungeanum* | MF039494 | MF070174 | MF070174 |
| ZB08 | *Z. bungeanum* | MF039495 | MF070132 | MF070203 |
| ZB09 | *Z. bungeanum* | MF039496 | MF070133 | MF070204 |
| ZB10 | *Z. bungeanum* | MF039520 | MF070125 | MF070202 |
| ZB11 | *Z. bungeanum* | MF039521 | MF070160 | MF070219 |
| ZB12 | *Z. bungeanum* | MF039522 | MF070126 | MF070216 |
| ZB13 | *Z. bungeanum* | MF039523 | MF070127 | MF070210 |
| ZB14 | *Z. bungeanum* | MF039519 | MF070124 | MF070201 |
| ZB15 | *Z. bungeanum* | MF039538 | MF070113 | MF070223 |
| ZB16 | *Z. bungeanum* | MF039537 | MF070114 | MF070224 |
| ZB17 | *Z. bungeanum* | MF039544 | MF070156 | MF070226 |
| ZB18 | *Z. bungeanum* | — | MF070161 | MF070227 |
| ZB19 | *Z. bungeanum* | — | MF070162 | MF070211 |
| ZB20 | *Z. bungeanum* | — | MF070163 | — |
| ZB21 | *Z. bungeanum* | — | MF070159 | MF070209 |
| ZB22 | *Z. bungeanum* | MF039497 | MF070128 | MF070212 |
| ZB23 | *Z. bungeanum* | MF039498 | MF070129 | MF070213 |
| ZB24 | *Z. bungeanum* | MF039499 | MF070130 | MF070214 |
| ZB25 | *Z. bungeanum* | MF039493 | MF070131 | MF070215 |
| ZB26 | *Z. bungeanum* | MF039541 | — | MF070181 |
| ZB27 | *Z. bungeanum* | MF039531 | — | — |
| ZB28 | *Z. bungeanum* | MF039542 | MF070151 | MF070218 |
| ZB29 | *Z. bungeanum* | — | MF070154 | MF070221 |
| ZB30 | *Z. bungeanum* | MF039539 | — | MF070205 |
| ZB31 | *Z. bungeanum* | MF039535 | MF070150 | MF070207 |
| ZA01 | *Z. armatum* | MF039508 | MF070165 | MF070187 |
| ZA02 | *Z. armatum* | MF039509 | MF070138 | MF070175 |
| ZA03 | *Z. armatum* | MF039510 | MF070139 | MF070188 |
| ZA04 | *Z. armatum* | MF039511 | MF070140 | MF070189 |
| ZA05 | *Z. armatum* | MF039512 | MF070141 | MF070190 |
| ZA06 | *Z. armatum* | MF039504 | MF070136 | MF070184 |
| ZA07 | *Z. armatum* | MF039502 | MF070145 | MF070177 |
| ZA08 | *Z. armatum* | MF039503 | MF070146 | MF070194 |
| ZA09 | *Z. armatum* | MF039506 | MF070147 | MF070196 |
| ZA10 | *Z. armatum* | MF039507 | MF070148 | MF070197 |
| ZA11 | *Z. armatum* | MF039505 | MF070164 | MF070217 |
| ZA12 | *Z. armatum* | — | MF070135 | MF070182 |
| ZA13 | *Z. armatum* | MF039530 | MF070152 | MF070208 |
| ZA14 | *Z. armatum* | MF039524 | MF070149 | MF070208 |
| ZA15 | *Z. armatum* | MF039543 | — | MF070222 |
| ZP01 | *Z. piperitum* | MF039500 | MF070115 | MF070180 |
| ZP02 | *Z. piperitum* | MF039501 | MF070116 | MF070179 |
| ZS01 | *Z. simulans* | MF039525 | MF070118 | MF070166 |
| ZS02 | *Z. simulans* | MF039526 | MF070157 | MF070167 |
| ZS03 | *Z. simulans* | MF039527 | MF070119 | MF070220 |
| ZS04 | *Z. simulans* | MF039528 | MF070120 | MF070168 |
| ZS05 | *Z. simulans* | MF039529 | MF070158 | MF070169 |
| ZM01 | *Z. micranthum* | MF039513 | MF070107 | MF070176 |
| ZM02 | *Z. micranthum* | MF039514 | MF070142 | MF070191 |
| ZM03 | *Z. micranthum* | MF039515 | MF070143 | MF070192 |
| ZM04 | *Z. micranthum* | MF039516 | MF070144 | MF070193 |
| ZM05 | *Z. micranthum* | MF039517 | MF070137 | MF070185 |
| ZSC01 | *Z. scandens* | MF039532 | MF070106 | MF070183 |
| ZSC02 | *Z. scandens* | MF039533 | MF070117 | MF070186 |
| ZMO1 | *Z. molle* | MF039518 | MF070105 | MF070178 |
| ZMO2 | *Z. molle* | MF039536 | MF070155 | MF070225 |
| ZAI | *Z. ailanthoides* | MF039534 | MF070108 | MF070195 |
| ZAC | *Z. acanthopodium* | — | MF070104 | — |
| ZEC | *Z. echinocarpum* | MF039485 | — | MF070228 |
| ZMU | *Z. multijugum* | MF039536 | — | MF070229 |
| ZD01 | *Z. dissitum* | — | MF070153 | — |
| ZD02 | *Z. dissitum* | MF039484 | MF070103 | MF070170 |
| ZOV | *Z. ovalifolium* | MF039486 | MF070134 | MF070230 |
