# Supplementary figures and images for: Internal transcribed spacer 2 (ITS2) barcodes: A useful tool for identifying Chinese Zanthoxylum
Source: Appl Plant Sci. 2018 Jun 15;6(6):e01157. doi: 10.1002/aps3.1157 (PMC6025816; doi:10.1002/aps3.1157)

APPENDIX S3. Gel images of ETS (top), ITS2 (center), and *trnH-psbA* (bottom) for some of the samples.

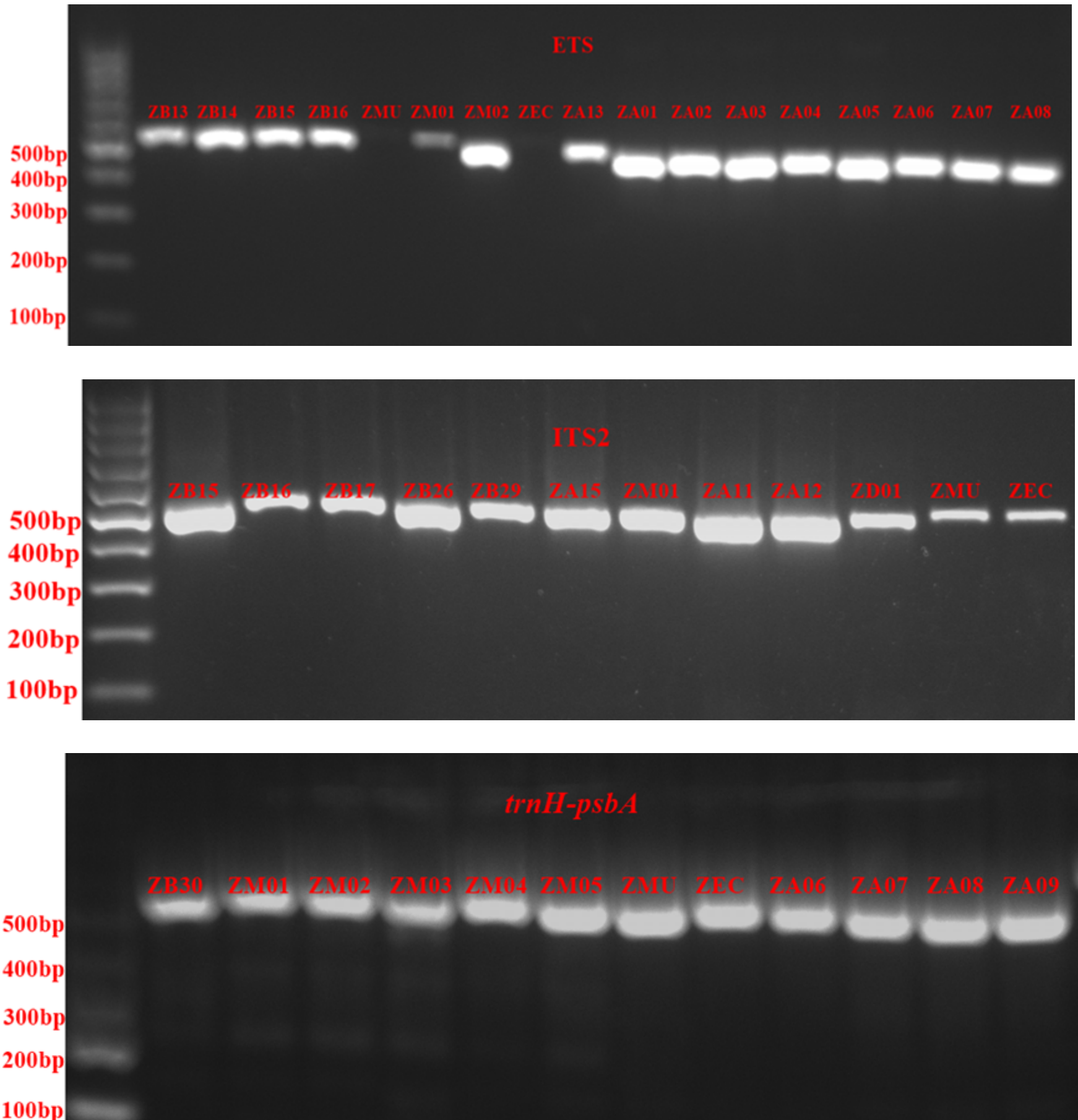

Supplement: Supplementary file 3 — Appendix S3 [file APS3-6-e01157-s003.pdf]
